# Investigation of Forward Tunneling Characteristics of InGaN/GaN Blue Light-Emitting Diodes on Freestanding GaN Detached from a Si Substrate

**DOI:** 10.3390/nano8070543

**Published:** 2018-07-18

**Authors:** Moonsang Lee, Hyunkyu Lee, Keun Man Song, Jaekyun Kim

**Affiliations:** 1 Korea Basic Science Institute, 169-148, Gwahak-ro, Yuseong-gu, Daejeon 34133, Korea; lms1015@kbsi.re.kr; 2 Department of Applied Physics, Hanyang University, Ansan 15588, Korea; lhk712@hanyang.ac.kr; 3 Device Development Department 2, Technology Development Division, 109, Gwanggyo-ro, Yeongtong-gu, Gyeonggi-do, Suwon-Si 16229, Korea; keunman.song@kanc.re.kr; 4 Department of Photonics and Nanoelectronics, Hanyang University, Ansan 15588, Korea

**Keywords:** InGaN/GaN LED, freestanding GaN, forward leakage current, conduction mechanism, tunneling

## Abstract

We report forward tunneling characteristics of InGaN/GaN blue light emitting diodes (LEDs) on freestanding GaN detached from a Si substrate using temperature-dependent current–voltage (*T-I-V*) measurements. *T-I-V* analysis revealed that the conduction mechanism of InGaN/GaN LEDs using the homoepitaxial substrate can be distinguished by tunneling, diffusion and recombination current, and series resistance regimes. Their improved crystal quality, inherited from the nature of homoepitaxy, resulted in suppression of forward leakage current. It was also found that the tunneling via heavy holes in InGaN/GaN LEDs using the homoepitaxial substrate can be the main transport mechanism under low forward bias, consequentially leading to the improved forward leakage current characteristics.

## 1. Introduction

Rapidly growing demand for energy saving and high luminescence of general illumination, and display, has encouraged a significant development of InGaN/GaN light-emitting diodes (LEDs) [1,2]. Owing to the lack of native bulk GaN, conventional InGaN/GaN LEDs were fabricated on foreign materials, such as Al_2_O_3_, SiC, and Si [3,4,5]. This inevitably evolves the generation of high dislocation density (10^8^–10^10^/cm^2^) in GaN-based devices, thus causing the deterioration of the device performance [6]. Although the introduction of freestanding GaN crystals as the substrate into InGaN/GaN LEDs can reduce the degraded device performance, its adoption into a GaN-based community has been inhibited, owing to their high fabrication cost and size issues. Recently, we demonstrated InGaN/GaN blue LEDs on freestanding GaN based on a Si substrate and showed their excellent device characteristics [7]. Despite its successful demonstration, the exploration on the forward leakage current of the homoepitaxial InGaN/GaN blue LEDs still remains unknown. Since the characteristics of forward leakage current in InGaN/GaN LEDs determine the device reliability, luminescence efficacy and electrostatic discharge resilience, and govern their continuous operation and soft and hard failure, understanding, identification and characterization of forward leakage current is of great importance to achieve high luminescence in the InGaN/GaN LEDs with excellent reliability [8]. In this paper, we investigate the forward leakage current characteristics of the InGaN/GaN LEDs using freestanding GaN detached from a Si substrate, via temperature-dependent current–voltage (*T-I-V*) measurements.

## 2. Materials and Methods

InGaN/GaN multi-quantum well (MQW) LEDs with peak emission wavelength of ≈440 nm were grown using MOCVD (Aixtron G3 2600, Aixtron, Herzogenraht, Germany) on 2-inch hydride vapor phase epitaxy (HVPE) freestanding GaN extracted from a Si substrate (LED I). The device structures and fabrication procedures were detailed in [7,9]. The LED epitaxial structure was composed of a Si-doped 3.5-µm-thick *n*-type GaN, 4 period multi-quantum well active layers consisting of 3-nm-thick InGaN well and 12-nm-thick GaN, and a Mg-doped 150 nm-thick *p*-type GaN layer. The dislocation density of LED I was estimated to be 1 × 10^6^ cm^−2^, which is confirmed by micro-photoluminescence (micro-PL) mapping (not shown in this paper). Additionally, conventional InGaN/GaN LEDs with corresponding structures and the peak emission wavelength were fabricated on 2-inch Al_2_O_3_ substrate (LED II), to compare the forward leakage current characteristics of LED I. LED II exhibits the dislocation density of 3 × 10^8^ cm^−2^. All the LED structures were fabricated with conventional lateral chip using a conventional photolithography, dry etching and metallization, which defined 350 µm × 350 µm chip size.

## 3. Results and Discussion

**Figure 1:**
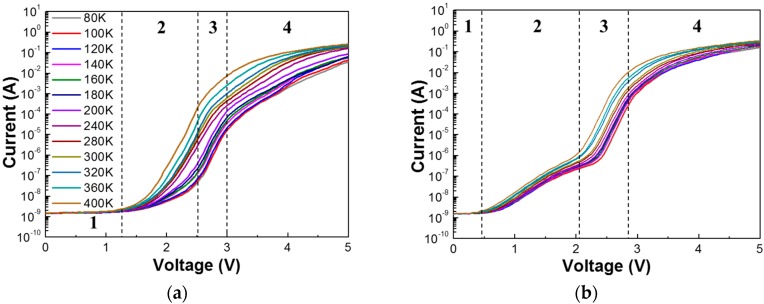
Temperature-dependent current–voltage (*T-I-V)* characteristics of (**a**) LED I and (**b**) LED II under forward bias from 80 K to 400 K.

Figure 1a,b shows the *T-I-V* characteristics of LED I and LED II in a logarithmic scale, respectively.

One can clearly observe that different segments in forward *I-V* characteristics in both LEDs are present, indicative of different dominant carrier transport mechanisms. It is well known that a tunneling mechanism is a dominant conduction process in low-bias regions [10]. Note that there is no abrupt initial increment of forward leakage current in *T-I-V* curves of LED I, followed by a gentle increase from about 1.3 V. On the other hand, a sudden increase of forward current from 0.3 V was clearly observed, accompanied by a fingerprint of a hump shape in *T-I-V* curves in LED II. We believe that this is attributed to a reduced threading dislocation density in LED I, compared to that in LED II. Furthermore, the slopes of *T-I-V* curves in LED I appear to be a function of temperature in the voltage ranges of 1.5‒3 V, suggesting an introduction of thermally activated current. This implies that diffusion and recombination currents start to dominate the transport mechanism, thus reflecting a high material quality of LED I [11]. On the contrary, the slopes of *I-V* characteristics of LED II are almost insensitive to temperature in the voltage range of 0.3‒3.5 V. This represents that a nondiffusion–recombination mechanism governs the tunneling transport in LED II [12]. At high voltage regimes (area 4 in Figure 1) of applied forward bias for both LEDs, the current is no longer increased exponentially with the applied voltage. This implies that series resistance-limited conduction starts to dominate the transport mechanism [13].

To get further insight into the tunneling mechanism and carrier entities for the LEDs, the forward *I-V* relationship is analyzed. The forward *I-V* characteristics in InGaN/GaN LEDs can be simply fitted by the Shockley’s equation [14],

(1) $$I=I_{0}exp\left(\frac{qV}{E_{T}}\right),$$

**Figure 2:**
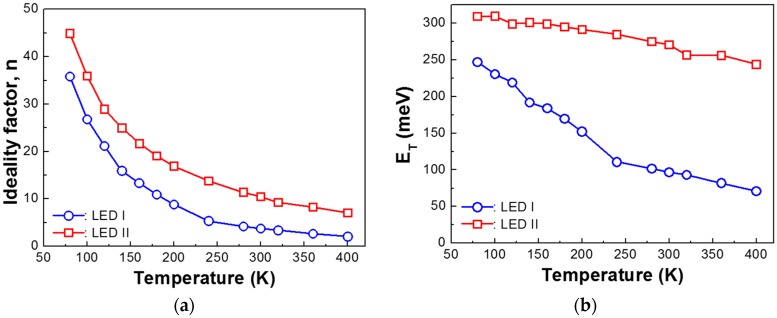

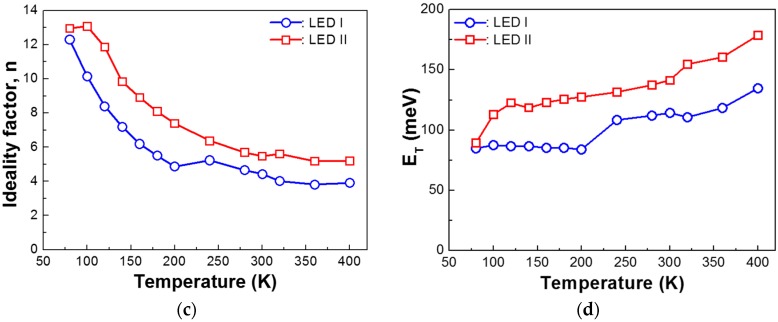
Ideality factor *n* and corresponding characteristic energy *E_T_* of the InGaN/GaN LEDs in the range of (**a**,**b**) low and (**c**,**d**) intermediate forward bias. Blue and red colors indicate LED I and LED II, respectively. The obtained values are deduced from Equations (1) and (2).

*E_T_* = *nkT*, (2)

where *I*_0_ is a pre-exponential factor, *q* is the elementary charge, *V* is the bias applied to LEDs, *E_T_* is the characteristic energy indicating the transparency of related energy barrier, *n* is the ideality factor, and *k* is the Boltzmann constant. The value of ‘*n*’ reflects the current transport mechanism in a device. When *n* is larger than 2, tunneling plays a dominant role on the conduction process. Recombination conduction dominates as *n* goes close to 2. On the other hand, if *n* is close to 1, the dominant transport mechanism is governed by diffusion current conduction [15]. Figure 2a–d illustrates the *n* and *E_T_* in low (area 2 in Figure 1) and intermediate (area 3 in Figure 1) bias regimes as a function of temperature for LED I and LED II, respectively. It is essential to note that the *n* of LED I is lower than that of LED II in all temperature ranges. Since a high ideality factor is related to defect-assisted tunneling current in InGaN/GaN LEDs owing to high threading dislocation [12,16], we can consider that enhanced forward leakage characteristics of LED I originate from higher crystallinity, compared to that of LED II. At low bias regions, the *n* and *E_T_* decrease with the increment of the temperatures for both LED I and LED II. It is interesting to note that *E_T_* of LED II reduced less compared to that of LED I. Note that a temperature-insensitive *E_T_* is typically considered as a feature of defect-assisted tunneling current in InGaN/GaN LEDs [12,17]. This is in excellent agreement that LED I exhibits reduced threading dislocation density. However, over intermediate injection voltage (Regime 3), the diode characteristics of both LEDs expressed temperature-dependent *E_T_*, and a significantly reduced ideality factor. We can infer that defect-assisted tunneling current is greatly suppressed in this regime. Furthermore, it should be noted that *n* of LED II starts at a higher factor compared to that of LED I. This is attributed to high dislocation density [12,16,18,19]. Beyond the intermediate bias regime, the carrier transportation of LEDs is influenced by an increasing voltage drop on the series resistance of *p*-GaN, as mentioned above [20].

To elucidate the tunneling entities in the InGaN/GaN LEDs, the characteristic energies were calculated, using the following equation [14],

(3) $$E_{T}=\frac{4qh}{\pi }\sqrt{\frac{N_{I}}{m^{*}\varepsilon_{s}}},$$

where *q* is the elementary charge, *h* is the Plank constant, *N_I_* is the reduced doping concentration at the space charge region (SCR), m* is the effective mass of the carriers, and *ε_s_* is the dielectric constant in SCR. *N_I_* can be computed as 2 × 10^17^/cm^3^ from the InGaN/GaN MQWs of LED I and LED II determined from the capacitance–voltage characteristics for low forward bias (not shown in this paper). The characteristic energy, *E_T_*, describes the barrier of multistep tunneling between defect sites at small forward bias, thereby allowing us to identify the tunneling entities at a specific bias regime. From Equation (3), we can obtain the values of *E_T_* for LED I and LED II in different bias regimes by substituting *N_I_*, and the effective masses of electrons and heavy holes (0.2*m*_0_ and 1.4*m*_0_ where *m*_0_ indicates effective mass of electron, respectively [21]). Obviously, heavy hole dominates the tunneling characteristics of LED I. Meanwhile, for LED II, the tunneling entities involve the electron and heavy hole at low and intermediate bias, as seen in Table 1. These results are consistent with the reported results [22]. It is noticeable that the *E_T_* computed from Equation (3) is different from the values of *E_T_* of LED I in the low bias regime of Figure 2. Since *N_I_* is weakly dependent upon the temperature variation [23], *E_T_* also would be weakly dependent on the temperature based on Equation (3). We speculate that this difference may be related to the carrier transport mechanism of LED I in the low bias regime, which is dominated by the thermally activated process, as mentioned above. A low thermal activation energy and high deep trap density in InGaN/GaN LEDs with low material quality may be the origin of the improved forward leakage characteristics, thus encouraging the carriers to move much more easily without the aid of thermal energy. On the sharp contrary, thermal energy in LEDs with high crystallinity can play an important role on the transport of charges, which requires one to overcome the high thermal activation energy and low trap density in electronic deep trap states, leading to significantly reduced leakage current. Therefore, it may be reasonable that LED I exhibits a temperature-dependent *E_T_* and the suppression of defect-assisted tunneling.

**Figure 3:**
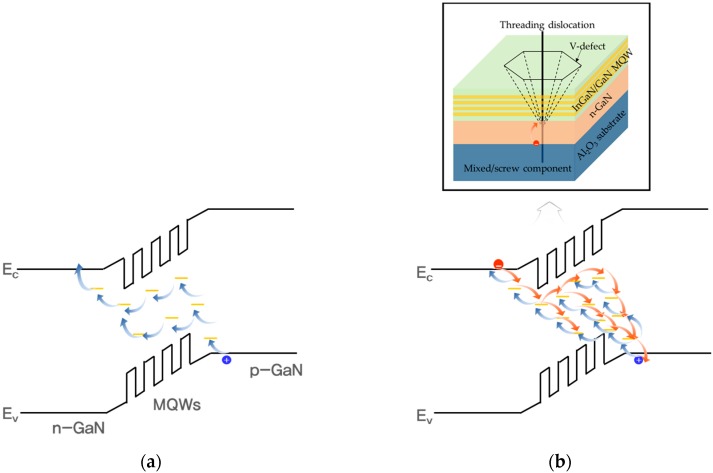
Energy band diagram with different V-shaped defect densities for (**a**) LED I, and (**b**) LED II. The inset depicts the carrier conduction along V-shaped defects with a component of mixed and screw dislocations. The red and blue solid dots represent the electrons and heavy holes, respectively.

According to Cao et al., the absence of V-defects with mixed and screw dislocation components in the homoepitaxial LED will encourage the prevention of electron tunneling to dislocations [25]. It is well known that electron tunneling is related to deep trap levels in the vicinity of mixed and screw dislocation, which is electrically and optically active in III-V alloys [22]. Suppose that the ratio of V-shaped defects with the component of mixed and screw in both LEDs is equal. This implies that the homoepitaxial LEDs exhibit reduced density of V-defects with a component of mixed and screw, compared to the LEDs using the heteroepitaxial substrates. It is evident that the homoepitaxial LEDs exhibit enhanced forward leakage current characteristics. Furthermore, improved forward tunneling characteristics of LEDs can be further confirmed by the tunneling entity, owing to its low mobility and large effective mass. Figure 3 shows the illustration of suggested tunneling phenomena of the InGaN/GaN LEDs. As mentioned above, heavy holes with large effective mass and low mobility in LED I would tunnel from *n*-side to *p*-side via defect sites in MQWs with low trap density. On the other hand, in the case of LED II, electrons and heavy holes would move across *n*- and *p*-sides through deep trap levels with high trap density. Considering this, we can conclude that the homoepitaxial InGaN/GaN LEDs using freestanding GaN substrate peeled from a Si substrate show significantly improved forward leakage current characteristics.

## 4. Conclusions

We investigated the forward tunneling characteristics of InGaN/GaN LEDs on freestanding GaN crystals extracted from a Si substrate, using *T-I-V* measurement. The forward *T-I-V* curves in InGaN/GaN LEDs using the homoepitaxial substrate exhibit different temperature-dependent slopes, indicating the transition of conduction mechanism from tunneling to diffusion and recombination transport. The theoretical calculations revealed that the forward tunneling characteristics of the LEDs using the homoepitaxial substrate are dominated by thermally activated processes owing to the improved material quality, and their tunneling entities, heavy holes, encourage the suppression of defect-assisted tunneling.

## Figures and Tables

**Table 1:** Summary of tunneling analysis of LED I and LED II. The obtained values are deduced from Equation (3).

Ref.	Substrate	Bias Region	*E_T_* (meV)	Dominant Tunneling Entity
This study	GaN	II, III	38.6	Heavy hole
This study	Sapphire	II	98.4	Electron
		III	38.6	Heavy hole
[22]	GaN	1.8–2.6	77	Heavy hole
[22]	Sapphire	0.3‒1.5	184	Electron
		1.9‒2.4	77	Heavy hole
[24]	Si	1.2‒1.8	128	Electron
		1.6‒2.5	56	Heavy hole
